# American Dental Association

**Published:** 1892-06

**Authors:** 


### AMERICAN DENTAL ASSOCIATION.

*August 7, 1891.—Fourth Day.—Morning Session.—(Continued.)*

MEETING called to order at 9.45.

Dr. Peirce offered a resolution, which was adopted, that the Association direct that sections belonging thereto shall, in the future, be prohibited from inviting individuals who are violating the code of ethics from holding clinics, or giving other exhibitions, in this organization. Section III. was then passed.

### SECTION IV.

### HISTOLOGY AND MICROSCOPY.

Dr. Abbott read the report of the section, in which he says that one paper has been referred to the section, by Professors Carl Heitzmann and Frank Abbott, of New York, entitled "Senile Atrophy of the Upper Jaw."

Following is an abstract of the paper:

With advancing age the human organism is reduced in size and weight,—the more so after the threescore years and ten have passed. The whole body shrinks, as well as the tissues. Age itself is a disease. The old Roman proverb says that the aged return to childhood, mentally and physically.

The question we have tried to solve is,—In what does this atrophy consist? Through the kindness of Dr. Paine, superintendent of the Insane Hospital in Westborough, Massachusetts, we came into possession of the upper jaw of a woman who died at the age of seventy-five years. It was placed in a half-per-cent. solution of chromic acid, in which it remained several weeks, for the purpose of softening the bony parts. The whole thickness of this jaw was not more than from four to five millimetres. It was perfectly smooth.

Let us consider the senile changes of the tissues involved in the formation of the upper jaw.

*First, epithelium.*—As mentioned before, the layer of stratified epithelium has been considerably reduced in bulk, although its constituent portions are still recognizable.

*Second, fibrous connective tissue.*—The senile changes are explained only by the fact that protoplasmic bodies lie between the fibrous connective tissue, which are traversed by a net-work of living matter. These bundles are possessed of life, therefore, as well as protoplasm itself.

*Third, blood-vessels.*—The fibrous connective tissues are freely supplied with blood-vessels during the ascending period of life, while they hold surprisingly few in advanced age. Smaller arteries show an augmentation of the nuclei to such an extent that the spindles of the muscle fibres become replaced by rows of minute globules.

*Fourth, nerves.*—Here and there we meet with medullated nerves, recognizable by their connection with other nerves which exhibit striking changes. Such changes are usually met with in the process of neuritis, but as this is not present in this instance, they must be attributed to senile changes.

*Fifth, cartilage.*—The appearance of hyaline cartilage in a senile jaw is a most surprising fact, going far to prove that rejuvenescence of the tissues takes place in old age.

*Sixth, bone-tissue.*—After individual teeth have been lost, their sockets after a while disappear, and when all the teeth are gone, the whole alveolar process fades away. This process is known by the term absorption, but we are not satisfied with the idea conveyed by this expression, for it is evident that a hard, tissue-like bone cannot be so easily disposed of. It must first be reduced to its protoplasmic condition, which can be disposed of more readily. The results of our studies in this direction fully justify us in stating that such changes do take place.

It is a fact that, in advancing age, the bone-corpuscles become less in number, the reason for which is that many of them are transformed into basis substance.

All tissues arise from protoplasm, and all tissues return to this embryonic state before their absorption. Origin and decline show a striking similarity, and what is between the threescore years and ten is struggle and change of the whole person, as well as of all its constituent parts, the tissues.

DISCUSSION.

*Dr. Hunt.*—It seems to me that this paper is a very valuable one. So far as I know, this is the first effort that has been made to demonstrate the destruction or wasting away of the bony tissue in the same line of investigation that has formerly been taken up in regard to the softer tissue. It is well established, I believe, that the process of destruction is a retrograde one, in full keeping with that of formation. This brings out the same idea as applied to bone-tissue. I have long been of the opinion that much of the investigation of this character was carried on more for the purpose of finding new laws than endeavoring to discover something like a universal law for the government of protoplasm both in building tissues and tearing them down. Therefore, I think this paper is very valuable in this respect. I presume it will lead to further investigation in that direction.

*Dr. Ingersoll.*—I will say that I have been more interested in Dr. Abbott's paper than any one I have read on histological subjects for many years. It preaches a practical fact, which every operator is brought in contact with nearly every day. We wonder what becomes of the tissue when we see it wasting away. What is absorption? I believe that we shall be obliged to come to this conclusion: that the functions which build up and afterwards tear down the tissues are the same. We see that in the case of absorption of the temporary teeth, and whatever the process may be, it is the same, I believe, in both instances. That of absorption is first a process of solution. There is no such thing as eating down the bone, but there is first a solution or return to a condition of solvency, and then absorption is an easy matter; but not until then. I must express my great satisfaction in other respects, in regard to histological observations in general. When we are called upon to explain a fact, and to seek for that explanation by the microscope, we must go right back to the embyronic condition. We are dealing with the perfected tissue, and that is a very different kind from the embryonic, in every instance. The brain-tissue of the infant is not that of the man. How then can I understand the brain-tissue of the man from microscopical observation of that of the child? Suppose I am a wood-worker; I want to know the nature of the oak. I ask my friend about it, and he says, "Here is an acorn; this is the embryonic oak." He has not given me what I want to know. I want to know why oak is more enduring than pine, or any other wood. I cannot understand from the embryonic condition at all. I cannot know very much about a hen from the egg. I do not want the embryo. We can best understand by going back to the first stages of tissue.

Here is a paper which is a thousand times more valuable than embryonic investigations. It has taken the mature tissue, and we want more of that kind of investigation. There is one point I did not quite understand. How is it, if the senile condition is a return to the embryonic, that the bone is so pliable, compared to what it is in childhood? If there is a return to the cartilaginous condition of the bone and protoplasm, why is it that the bone is so fragile in old age?

*Dr. Abbott.*—I will endeavor to answer that question. The bone-corpuscles, of which there are many in the bones of young and middle-aged people, as they grow older, are converted into their embryonic state; and those medullary bodies take in lime salts, which eventually fill the bone-corpuscles. Thus it becomes solid, as compared with what it originally was. It is simply converting the bone-corpuscles into bone itself, by the processes described in the paper.

Section V.

### MATERIA MEDICA AND THERAPEUTICS.

Dr. Harlan reports that Section V. has only one paper, by Dr. Marshall, of Chicago, entitled, "Electricity as a Therapeutic Agent in the Treatment of Hyperæmia and Congestion of the Pulp and Peridental Membrane."

The following is an abstract of Dr. Marshall's paper:

I have chosen as the special topic of this paper two out of several important pathological conditions of the teeth, to which the various forms of treatment by electricity may be beneficially applied.

Hyperæmia and congestion of the dental pulp from caries, thermal shock, chemical and mechanical irritants, and traumatic injuries, etc., resulting in odontalgia, are among the most common of the diseased conditions found in the oral cavity, and many times the most difficult to control by the methods of treatment usually adopted without devitalization of the pulp.

The object, of course, in the treatment is to relieve the congested condition of the blood-vessels and to preserve the vitality of the pulp, and to arrest the inflammatory symptoms short of the suppurative process, or of the formation of adventitious tissue or new growths.

How these much-to-be-desired conditions can be obtained is a question that has often troubled the mind of the thoughtful dentist; and while I do not claim to have made a new discovery, I am confident that the galvanic current, if judiciously used, will prove to be a valuable aid in the treatment of certain forms of inflammation of the pulp and peridental membrane.

The pulp of the right first superior bicuspid in the writer's mouth becomes congested. It was determined to try the depleting effect of the positive galvanic current. The positive pole of the continuous galvanic current was applied to the tooth, and the negative pole to the carotid triangle of the neck on the same side. The strength was graduated to my ability to bear it without discomfort, and the poles were allowed to remain in position for about a half-hour. At the end of ten minutes there was a marked improvement in the symptoms, and at the end of the half-hour all discomfort in the tooth had disappeared.

The marked success which followed the treatment of this tooth has led me to adopt the same in several similar cases, and all but one of which have responded to my entire satisfaction.

In the treatment of pericementitis, not caused by septic poisoning from a devitalized pulp, it is many times of very great benefit. In these cases the positive pole should be applied to the gum over the roots of the affected tooth.

In a former paper I have called attention to the prevalence of hyperæmic odontalgia frequently accompanying pregnancy. I would suggest the local application of the positive galvanic current to the affected teeth.

As a rule, one treatment in twenty-four hours is all that will be required.

As a means of diagnosis in obscure cases of the vitality or non-vitality of the dental pulp, I know of nothing so sure to demonstrate to a positive certainty these conditions as the electrical currents, both the galvanic and the faradic. In the more obscure cases the faradic is superior to the galvanic. I also believe that the electric current will serve to demonstrate the presence of low grades of inflammation of the tooth-pulp. The diseased tooth will give a more active response to the current than will the healthy tooth.

DISCUSSION.

*Dr. Frederichs.*—Is there not some mind-cure in this? You may laugh, but there is some truth in my remarks. The mind has a great effect on the result of cure. The reason I arose to speak about it is that it will cure sometimes. I had that illustrated in myself. I have been a sufferer from lumbago. The first time electricity cured it in about two weeks; but we know by the general tendency that I would have gotten well in about six weeks without anything, because all diseases have a tendency to cure themselves. I know of one instance where electricity was applied by a physician in order to relieve toothache, because he was a sort of monomaniac on the subject. He even had his battery brought from Europe. I know positively that he applied it to relieve his patients of toothache. He did not succeed, and he finished by the injection of an extra grain of morphine. I believe electricity is a good thing, and, if I adopt anything that has been illustrated here, I shall certainly apply that relief to patients in my practice; but what I wanted to add to my remarks was that the remedy, when applied a second time in my own case, had no effect.

*Dr. Abbott.*—The little instrument that is passing round, exhibited by Dr. Marshall, reminds me that some years ago I made one of a similar kind, only the back end of it was a bow instead of being brought together by a rubber arrangement as that is. At each end there was a cup, facing each other, the same as that. It was about three-quarters of an inch in diameter and nearly half an inch in depth. In each of these I packed a sponge, and saturated it with this mixture:

℞ Tinct. aconiti rad.,
Chloroformi,
Alcoholis, āā ℥i;
Morphinæ sulph., gtt. xii. M.
Sig.—To be used as a local application.

I used it for extracting teeth. I held it against each side of the tooth; and you can extract teeth as fast as you please successfully. It just occurred to me, and I thought I would make mention of it.

*Dr. Frederichs.*—What is the object of adding alcohol?

*Dr. Abbott.*—All those things tend to one thing,—anæsthesia. Some gentlemen understood that I used electricity with this. I did not; but I think electricity would intensify the action of any material of this kind very much. By using it you can produce the effect in a very few moments. Surgeons use the electrodes for forcing an antiseptic into such place as they wish.

*Dr. Horton.*—In carrying out this idea of the application of electricity to reduce inflammation, I would say that my son has prepared an apparatus by which he overcomes all sensitiveness in the dentine in excavating preparatory to filling teeth. He is not in a position to make any illustrations here, but I hope that at the next meeting of this society he will be there to show you what he can do, so far as excavating teeth by electricity is concerned. He has used it on perhaps one hundred patients very successfully. I hope he will soon be able to put it into the hands of any one of ordinary intelligence, and the dentist will not be obliged to have a bottle of medicine standing next to him to relieve sensitiveness of dentine.

*Dr. Hunt.*—I would like to ask the essayist exactly what he means by the use of the term "embolus" in relation to the pulp?

*Dr. Marshall.*—I mean by the term what is generally understood by dentists. I mean a clot of blood which has formed, and that the arterial supply has been plugged up, probably by some little globule or fibre, or by the formation of a blood clot.

*Dr. Frederichs.*—I certainly was pleased with Dr. Marshall's suggestion. We all know that electricity—although it has been used medicinally for some time—is still in an experimental state. As far as we know, there is really no definite ideas upon which to base its application. It requires a great deal of original investigation of these matters to get them perfect. I do not believe in tabooing anything that has the least show of being productive of good. In regard to emboli, I cannot conceive how, in a case of that kind, electricity would be effective.

*Dr. Marshall.*—It did not do any good.

*Dr. Morgan.*—Perhaps I know less about electricity than any man in the audience, but for the last four or five years I have had great difficulty in my locomotion. I have a bad case of rheumatism, with a touch of gout. A man came to me one day with a small piece of wire with two bowls attached to it. One of these he proposed to attach to my person. I was then walking with great difficulty, with a crutch and a stick, and I had to have both these aids to get across the floor. I put it on, and was enjoined not to take any medicine. It has stood by the side of my bed, and I have used it ever since. In about a week or ten days I began to sleep a little better, and the pains began to subside, and then in about three months, wearing it every day and every night, that foot began to get a little better. I wore it almost constantly from that time up to the present, and am able to walk alone now. I am free from pain, can sleep like a baby, my digestion is good, and the trouble with my heart, from which I suffered for a long time, has passed entirely. Here I am in my seventy-third year, and I think I will be able to throw away the crutches. That is what I know about electricity. Although I am not restored entirely, I feel that it has done me a great deal of good. I could not fix its value in dollars. I give that as an illustration of the value of this agent. I do now know what it is. Our experts at Nashville took it down to examine it, and they say there is no electricity whatever in that instrument. It does some very wonderful things. In one case it caused the healing up of some ugly ulcers that were of at least thirty-five years standing, in seven weeks.

*Dr. Abbott.*—I would like to emphasize what has just been said by Dr. Morgan. He has had some very wonderful results from it. You could not buy that instrument from him for any amount of money. I think the man who makes them lives in Birmingham, Alabama.

*Dr. Shepard.*—I merely want to say that I have no doubt, from the testimony of the most reliable people that lived, that there is at the bottom of these cases, and many others, the same great principle of mind influence. We all know a pretended medicament is frequently as efficacious as a true one. When the most intelligent and educated people in our communities are firm believers and practitioners of Christian Science, and the kindred things, we must admit that they are not lunatics, but that there is some principle in it which is not yet fully understood. We are on the threshold of some great truth, which misleads us when we do not understand it, and makes us think that the medicines we use are good and useful. We might as well take these things into account and avail ourselves of them in our practice, and when better understood, they will perhaps explain the multitude of clever things that happen.

*Dr. Marshall.*—This discussion has taken a very wide range, and it would take me a good while to answer all the points. Dr. Frederichs and Dr. Shepard have thrown out the suggestion that perhaps this idea of electricity being beneficial in the treatment of disease was owing to the influence of the mind, that it was partly a mind-cure. Now, these gentlemen are not serious, certainly, when they make these statements, because we know we can reduce certain forms of tumors in the human body by the action of the positive galvanic current. That point is settled. It stimulates the absorbents, and in that way the tumor is carried away. There is another point that I brought out in my paper. That is the production of hyperæmia on the surface of the body by the application of the positive galvanic current, and the reverse—anæmia—by the negative current. You can drive the blood out of a part of the body, or you can bring it in. Any one can test that, if he has a battery. When I first commenced these treatments, I used my first finger as the electrode. I could not leave it there more than about five minutes before the current decomposed the water and the secretions under my nail, until it began to smart so that I could not stand it.

I might have recited several cases in support of my theory. I thought if I gave you one that was successful and one that was a failure, it would illustrate the point. A lady in Chicago, who is a physician, living on the north side, and who is one of the most talented women in our city, had this experience; in the excavation of a cavity in a bicuspid tooth, one of the horns of the pulp was slightly exposed, and her dentist filled it with oxyphosphate cement. It was not odontalgia; it was that low, grumbling movement that made her think every moment the tooth would begin to ache. That was a condition of hyperæmia. I suggested electrical application, and she said she had no faith in that. I persuaded her to use it, and in less than half an hour that tooth was quiet. She came back and told me that the tooth had troubled her again; she said she had taken some hot coffee, and that had annoyed the tooth. I tried the current again for three days, one sitting a day, and it has ended. She has come back at different times, and the pulp is still alive. I hope to save it, too. It is in cases where you find the first stages of hyperæmia, where it will probably go on and end in the destruction of the pulp, if let alone, that this is valuable. In those cases it will relieve the hyperæmia and save the tooth.

Dr. Abbott spoke of the introduction of medicaments into the pulp. There are experiments being tried in regard to that. I have never tried it, but before I left home that matter was brought up by me in a conversation with Dr. McIntosh. We applied some solutions which we used, to see if we could get the medicament into the tissues, so as to get a more rapid action. I thought this could be done, and I proposed trying it.

Subject passed.

Dr. Truman stated that Dr. Stebbins had his son present, to show the action of nitrate of silver on deciduous teeth.

*Dr. Stebbins.*—I will state the manner of procedure that I have had under consideration for several years, and that was to arrive at some method by which we might save the temporary teeth without cutting or hurting them. I have taken the crystals of nitrate of silver for the work, and pulverized them, because there is less impurity in them than in the sticks. The nitrate of silver forms a compound with the albumen in the fibres. This compound is dissolved by very few substances. I start out with that. The salts are very hostile to microbes, almost as much so as mercury salts. The time to take it is when the teeth begin to decay. I have found it effective on labial and buccal surfaces of teeth. When this boy was three or four years old, he complained that he could not brush his teeth, because they hurt him. I tried the application to the buccal surfaces. That was six years ago last March. They have not been touched since. The approximal surfaces were treated in 1887. I have touched those two or three times. I have treated many teeth not only temporary but permanent. I pulverize the agent in a mortar, and then take a wooden point, and having it moistened enough to have the crystals stick to it, apply it to all the parts of the tooth. Do not be afraid of getting too much in. In February and March, 1888, I took a number of children out of the primary schools and asked them to have their teeth treated in this manner. In May, 1891, I examined one hundred and twenty-four cavities, and found eighty-four where the treatment was successful.

MISCELLANEOUS BUSINESS.

A vote of thanks was offered to the Local Committee of Arrangements, and especially to Dr. A. C. Rich, for the efficient service rendered in making the stay of the Association a pleasant one.

Final reading of the minutes.

Drs. McKellops and Abbott were appointed to conduct the newly-elected president to the chair.

*Dr. Walker.*—Mr. President and members of the American Dental Association,—I feel deeply the honor you have conferred upon me in electing me as your presiding officer for the coming year, and I will promise to fill the position to the best of my ability.

On motion of Dr. Abbott, Dr. Corydon-Palmer was allowed ten minutes to proceed with his remarks on operative dentistry.

*Dr. Corydon-Palmer.*—For the past few years I have not attended the meetings of this society. The American people, as a nation, are being disgraced by having their mouths filled with gold. You will see a whole row of inferior incisors capped with gold. I want to urge upon you to stop this practice; it is discreditable; it is unnatural; it is unsightly; it is bad taste. Try to get along without it.

What I have been grieved about most is the treatment of children's teeth, where there has been necrosis, and, as far as I know, there is not enough knowledge upon that subject. You do not take pains enough to instruct the students in this branch. I have been shocked and grieved in the colleges where I have been to see the students at once extract the teeth. You must not do it. There is more harm done to the development of the teeth by interfering with the deciduous teeth than in any other way. You get deformity and want of development and irregularity. You must not extract them. Treat them; take care of them.

Those two subjects have impressed me so strongly that I felt I must say something about them. I hope each one will, as far as possible, endeavor to influence others to not interfere with children's teeth, and where a young mother or any person brings a child to you, treat the teeth instead of extracting them.

*Dr. Floyd.*—I would like to say one word. I came here from Kansas as one of the delegates, and as a member of this society, and I feel I have learned a great deal since I have been here. I have heard some very valuable papers, and I hope they will go out to the community as bright and shining lights.

